# Supplementary material for: Pedestrians’ Perception of Pedestrian Bridges—A Qualitative Study in Dar es Salaam
Source: Int J Environ Res Public Health. 2022 Jan 22;19(3):1238. doi: 10.3390/ijerph19031238 (PMC8835090; doi:10.3390/ijerph19031238)
Supplement: Supplementary file 1 [file ijerph-19-01238-s001.zip › ijerph-1531956-supplementary.pdf]

Box S1: Interview guide

Tell me a little bit about yourself.

Explore age, school, work, profession, and family.

Can you tell me what your attitude is to the bridge?

- What do you think about/know about its design and construction?
- What is your opinion of the structure of this pedestrian bridge?
- Why do you think they built the bridge here?
- Which criteria do you use in choosing the climbing ramp when accessing this bridge?

What is your comment on the interaction between vehicles and pedestrians along the bridge?

- Explore. Road signs?
- Explore. Junction near by the bridge?
- Explore. Speed of the vehicles?

Tell me how you feel about your personal safety and security when using the bridge both in the afternoon and in the night?

How does the presence of photographers influence your decision to use the bridge?

Tell me the role of traffic police alongside the pedestrian bridge?

- What is your opinion on the role of traffic police when guiding vehicles and pedestrians at a place which has a pedestrian bridge?
- What is your opinion on the involvement of road users when constructing pedestrian bridges?
- What suggestions do you have on how that involvement can be improved?
- Are there specific groups of people that need to be involved before a bridge is constructed?

Do you have any other comments?
